# Demographics and Health Characteristics Associated With the Likelihood of Participating in Digitally Delivered Exercise Rehabilitation for Improving Heart Health Among Breast Cancer Survivors: Cross-Sectional Survey Study

**DOI:** 10.2196/51536

**Published:** 2024-12-16

**Authors:** Tamara Jones, Lara Edbrooke, Jonathan C Rawstorn, Linda Denehy, Sandra Hayes, Ralph Maddison, Aaron L Sverdlov, Bogda Koczwara, Nicole Kiss, Camille E Short

**Affiliations:** 1 Melbourne Centre for Behaviour Change, Melbourne School of Psychological Sciences, The University of Melbourne, Redmond Barry Building, Tin Alley, Melbourne, 3010, Australia, 61 409498820; 2 Department of Physiotherapy, Melbourne School of Health Sciences, The University of Melbourne, Melbourne, Australia; 3 Department of Health Services Research, The Peter MacCallum Cancer Centre, Melbourne, Australia; 4 Institute for Physical Activity and Nutrition, Deakin University, Melbourne, Australia; 5 Cancer Council Queensland, Brisbane, Australia; 6 Newcastle Centre of Excellence in Cardio-Oncology, The University of Newcastle, Hunter Medical Research Institute, Calvary Mater Newcastle, Hunter New England Health, Newcastle, Australia; 7 Flinders Health and Medical Research Institute, Flinders University, Adelaide, Australia

**Keywords:** digital health, breast cancer, exercise, rehabilitation, cardiotoxicity, demographic, cancer survivor, exercise rehabilitation, home-based program, pathologic process, radiation, physical phenomena, heart care, cardiovascular disease, diagnosis, cross-sectional study, chronic disease, statistics

## Abstract

**Background:**

Strong evidence supports the benefits of exercise following both cardiovascular disease and cancer diagnoses. However, less than one-third of Australians who are referred to exercise rehabilitation complete a program following a cardiac diagnosis. Technological advances make it increasingly possible to embed real-time supervision, tailored exercise prescription, behavior change, and social support into home-based programs.

**Objective:**

This study aimed to explore demographic and health characteristics associated with the likelihood of breast cancer survivors uptaking a digitally delivered cardiac exercise rehabilitation program and to determine whether this differed according to intervention timing (ie, offered generally, before, during, or after treatment). Secondary aims were to explore the knowledge of cardiac-related treatment side-effects, exercise behavior, additional intervention interests (eg, diet, fatigue management), and service fee capabilities.

**Methods:**

This cross-sectional study involved a convenience sample of breast cancer survivors recruited via social media. A self-reported questionnaire was used to collect outcomes of interests, including the likelihood of uptaking a digitally delivered cardiac exercise rehabilitation program, and demographic and health characteristics. Descriptive statistics were used to summarize sample characteristics and outcomes. Ordered logistic regression models were used to examine associations between demographic and health characteristics and likelihood of intervention uptake generally, before, during, and after treatment, with odds ratios (ORs) <0.67 or >1.5 defined as clinically meaningful and statistical significance a priori set at *P*≤.05.

**Results:**

A high proportion (194/208, 93%) of the sample (mean age 57, SD 11 years; median BMI=26, IQR 23‐31 kg/m^2^) met recommended physical activity levels at the time of the survey. Living in an outer regional area (compared with living in a major city) was associated with higher odds of uptake in each model (OR 3.86‐8.57, 95% CI 1.04-68.47; *P*=.01‐.04). Receiving more cardiotoxic treatments was also associated with higher odds of general uptake (OR 1.42, 95% CI 1.02-1.96; *P*=.04). There was some evidence that a higher BMI, more comorbid conditions, and lower education (compared with university education) were associated with lower odds of intervention uptake, but findings differed according to intervention timing. Respondents identified the need for better education about the cardiotoxic effects of breast cancer treatment, and the desire for multifaceted rehabilitation interventions that are free or low cost (median Aus $10, IQR 10-15 per session; Aus $1=US $0.69 at time of study).

**Conclusions:**

These findings can be used to better inform future research and the development of intervention techniques that are critical to improving the delivery of a digital service model that is effective, equitable, and accessible, specifically, by enhancing digital inclusion, addressing general exercise barriers experienced by chronic disease populations, incorporating multidisciplinary care, and developing affordable delivery models.

## Introduction

Advances in cancer treatment and care have contributed to improved breast cancer–specific survival rates and a growing population of long-term breast cancer survivors [1,2]. Among this group, the late effects of specific cardiotoxic cancer therapeutics can adversely impact cardiovascular health and, consequently, both quality of life and survival [1]. Cardiovascular disease is now the leading cause of morbidity and mortality among long-term breast cancer survivors [1,3]. Notably, cardiovascular mortality rates are higher in women with breast cancer compared with the general population (hazard ratio 1.3, 95% CI 1.0-1.7) [3]. The development of cardiotoxicity is further exacerbated by the shared underlying risk factors for developing both cancer and cardiovascular disease, such as obesity and physical inactivity [4].

Participating in exercise rehabilitation may help to improve cardiovascular health among breast cancer survivors. Obesity, lower levels of cardiorespiratory fitness, and lower physical activity levels are known risk factors for cardiovascular disease and cancer [5-7]. There is strong evidence to support that exercise rehabilitation following cardiovascular disease can reduce morbidity and mortality [8,9]. The evidence supporting the benefits of exercise following breast cancer is also strong, with improvements in fitness, quality of life, and physical functioning well established from randomized controlled trials [6,10]. Despite the exacerbated risk of cardiovascular disease as a consequence of breast cancer treatment and the clear potential for exercise rehabilitation in this context, providing comprehensive exercise support to people with breast cancer in a safe, feasible, and effective way remains a major service delivery challenge [11,12].

Historically, clinic-based, face-to-face delivery of exercise therapy or prescription has been considered the gold standard and has been the delivery mode most commonly assessed in exercise oncology trials [10,13]. However, lessons from exercise rehabilitation in the cardiac setting highlight uptake and retention issues that come with clinic-based, face-to-face exercise delivery. Specifically, fewer than one-third of Australians who are referred to exercise rehabilitation complete a program following a cardiac diagnosis [14].

Technological advances make it increasingly possible to embed real-time supervision, tailored exercise prescriptions, behavior change, and social support into home-based programs [15]. These techniques may lead to higher exercise adherence, which is a key determinant of efficacy [15]. Remote monitoring and personalized feedback have also been reported by participants with breast cancer to be preferred attributes of technology-supported interventions [16]. A recent randomized controlled trial involving participants with coronary heart disease demonstrated the non-deidentified inferiority of a remotely-delivered cardiac rehabilitation intervention compared with standard face-to-face rehabilitation [17]. Furthermore, those in the remotely-delivered group demonstrated greater improvements in longer-term behavior change and the intervention cost significantly less to deliver [17]. It seems plausible this delivery approach could prove beneficial in an exercise oncology setting.

There is, however, a risk that those who are least likely to uptake digital rehabilitation interventions may include those who could benefit the most. Specifically, while a digital-delivery approach could address cost and access barriers (which are of particular concern for those with lower income or who live in regional/rural areas [18]), these benefits may be counterbalanced by poor internet availability and digital literacy for these same groups [18]. Digital literacy refers to “the capabilities and resources required for individuals to use and benefit from digital health resources” [19]. Digital literacy is, therefore, an interaction between systemic and individual factors, and is inclusive of several domains, including “the ability to actively engage with digital services”, “digital services that suit individual needs”, “access to systems that work,” and “engagement in our own health”. Additionally, barriers to uptaking digitally delivered exercise rehabilitation may also be reflective of general exercise barriers experienced by other chronic disease populations. A greater understanding of the individual characteristics likely to predict uptake is needed to proactively address potential inequalities or barriers that the use of this delivery model may bring.

Therefore, the aim of the current study was to explore individual demographic and health characteristics associated with the likelihood of uptake of a digitally delivered exercise rehabilitation intervention among breast cancer survivors, and to explore whether uptake differs according to intervention timing, that is, whether it is offered (1) generally, (2) before, (3) during, and (4) after treatment. Secondary aims were to explore the knowledge of cardiac-related treatment side-effects, the use of and interest in fitness trackers and apps, exercise behavior, additional intervention topics of interest, and service capabilities.

## Methods

### Ethical Considerations

This cross-sectional study involved the completion of a web-based survey between March and December 2020 in Australia. Ethics approval was sought and obtained from the University of Melbourne Human Research Ethics Committee (1955472). Informed consent was obtained from all subjects involved in the trial and all data were deidentified for privacy and confidentiality. Participants had the opportunity to leave contact details if they wished to receive a Aus $5 gift card for completing the survey (Aus $1=US $0.69 at the time of study).

### Participants and Procedure

Participants were recruited via paid Facebook advertisements (run during March and June 2020) and cancer-specific research registries (ie, Breast Cancer Network Australia membership list July 2020; National Breast Cancer Foundation Register4 December 2020). Participants were eligible if they were diagnosed with breast cancer in the past 5 years, were aged 18 years and older, able to answer the survey in English, and resided in Australia. All potentially eligible participants were directed to a web-based survey where they could read the study information sheet, confirm eligibility, provide informed consent, and complete the survey. At the end of the survey, participants could leave contact details if they wished to receive the Aus $5 gift card (US $3.45) [20], receive a summary of the results, or be contacted for future research opportunities. The survey was conducted via Qualtrics and survey items were informed and reviewed by 2 women with breast cancer and the multidisciplinary research team, including physiotherapists, behavioral scientists, a dietitian, a cardiologist, and a medical oncologist.

### Outcomes of Interest

#### Primary Outcome of Interest

##### Overview

The primary outcome of interest was the likelihood of uptake of a digitally delivered exercise rehabilitation intervention among breast cancer survivors, and to explore associated demographic and health characteristics. Assessment methods are described below.

##### Likelihood of Uptake of Digitally Delivered Cardiac Exercise Rehabilitation

The likelihood of uptake was assessed using purpose-built survey items on a 5-point Likert scale ranging from “not at all likely” to “extremely likely.” Participants were asked to rate how likely they would be to participate in a program generally, and before, during, and after treatment. The question stem included contextual information, including possible design features and anticipated benefits (Document S1 in Multimedia Appendix 1).

##### Demographics and Health Information

Participants reported their age, height, weight, postcode (remoteness determined based on Australian Statistical Geography Standard [21]), marital status, education, employment, if they were of Aboriginal or Torres Strait islander origin, and languages spoken at home. Participants rated their general health (poor to excellent) and whether they had been diagnosed with high cholesterol, high blood pressure, diabetes, congestive heart failure, heart attack, stroke or transient ischemic attack, depression or anxiety, or another health condition (used to calculate a study-specific comorbidity index; range=0‐8). Breast cancer history information was self-reported, including stage at diagnosis, treatments received, time since diagnosis, and current disease state. Participants who had received a treatment associated with cardiotoxicity (chemotherapy, radiotherapy, or human epidermal growth factor receptor 2 [HER-2] therapy), had ≥2 risk factors for cardiovascular disease (ie, age>60 years, BMI>30, hypertension, diabetes), or self-reported compromised cardiac function were categorized as having a high risk of cardiotoxicity based on the American Society of Clinical Oncology Guidelines [1].

### Secondary Outcomes of Interest

#### Overview

Secondary outcomes of interest included knowledge of cardiac-related treatment side-effects, use of and interest in fitness trackers and apps, exercise behavior, additional intervention topics of interest, and service capabilities. Assessment methods are described below.

#### Knowledge of Cardiac-Related Treatment Side Effects

Self-reported knowledge of cardiac-related side effects from cancer treatment was assessed using a single item on a 5-point Likert scale (“not at all knowledgeable” to “extremely knowledgeable”).

#### Use of and Interest in Fitness Trackers and Apps

Participants were asked whether they had used a fitness tracker or smartphone app to monitor exercise or other health behaviors. Where participants reported having previously used a smartphone app, they were prompted to specify which apps they had used.

#### Exercise Behavior

Exercise behavior was assessed using the Godin Leisure Time Exercise Questionnaire (GLTEQ) [22] and a purpose-built resistance-training item using the same format. For the GLTEQ, participants indicated how many times on average they spent doing strenuous, moderate, and mild or light exercise for ≥15 minutes during a 7-day period. Activity frequencies were multiplied by 9, 5, and 3 metabolic equivalents, respectively, and summed to create a total activity score. Participants were considered sufficiently active to obtain health benefits if they had a total activity score ≥24. Scores between 14 and 23 units were considered moderately active, and those <14 units as insufficiently active [23]. Using the same question stem, participants indicated how many times per week they spent doing resistance (strength) activities (eg, weights, yoga). Participants were classified as meeting the guidelines if they reported doing ≥2 sessions during a typical 7-day period [24].

#### Additional Intervention Interests

A single survey item assessed interest in 12 other intervention topics of interest relating to health behaviors and symptom management.

#### Service Capabilities

The dollar amount (Aus $) participants would be willing or able to pay per session was assessed with a single survey item.

### Data Analyses

Descriptive statistics were calculated for all survey items. Ordered logistic regression models were used to examine associations between demographic and health characteristics and the likelihood of intervention uptake. Four models were estimated: uptake generally, uptake before treatment, uptake during treatment, and uptake after treatment. Given prior research acknowledging the potential importance of demographics and health status in explaining health service–related inequalities [25], all collected demographic and health status variables were screened for inclusion. Variable selection was refined based on bivariate analyses, with the outcome variable regressed on each demographic and health variable using all available data. Variables that were statistically significant (*P*≤.05) or those of potential theoretical importance to digitally delivered services (eg, location) were selected for inclusion in the multivariate model [26,27]. The potential for multicollinearity was assessed by examining associations between remaining variables (using χ^2^ tests, correlation matrices, and regression analyses as appropriate) to determine variables to be retained in the final model. At each step, variables were retained if they were statistically significant (*P*≤.05) or clinically relevant (odds ratio [OR] <0.67 or >1.5) [28,29]. The variable with the highest *P* value was removed from the model (assuming it satisfied the elimination criterion, ie, a *P*>.05 or OR <0.67 or >1.5). The procedure ceased when there were no variables in the model that satisfied the elimination criterion (ie, all variables in the model had a *P*≤.05 or OR <0.67 or >1.5). All analyses were complete case analyses and conducted using SPSS Statistics for Windows (version 28.0; BMI).

## Results

### Participant Flow

Overall, 250 eligible individuals started completing the survey, with 87% (n=218) completing outcomes of interest (ie, the likelihood of participating in a remotely-delivered exercise intervention generally, and at different time points) and 83% (n=208) completing all demographic and health variables. There were no significant differences in likelihood ratings between individuals with and without missing data for patient characteristics. All analyses were performed on a complete case basis (N=208).

### Participant Characteristics

Participant characteristics are presented in Table 1. Participants had a mean age of 57 (SD 11) years and a median BMI of 26 (IQR 23-31) kg/m^2^. Approximately two-thirds resided in a major city (132/208, 64%) and 3 in 4 participants were married or living with a partner (153/208, 74%). The majority of participants had a university degree (137/208, 66%) and engaged in regular exercise (194/208; 93%). Over half of the participants surveyed (120/208, 58%) reported “little” to “no” knowledge of potential cardiotoxic effects of treatment, with just under half (94/208, 45%) being classified as being at high risk of cardiotoxicity. In terms of use of and interest in technology for fitness, 62% (129/208) of participants reported having previously used a fitness tracker and a further 30% (63/208) expressed an interest in trying one. Fewer participants had previously used a smartphone app (87/208, 42%), and of the participants who had not used a smartphone app, three-quarters (92/208, 76%) were open to trying. The types of apps most commonly reported as having previously been used included in-built health apps (Apple health and Samsung health), apps linked to specific fitness trackers (eg, Fitbit and Garmin Connect), popular fitness apps (Map My Walk, My Fitness Pal, Strava, and Run Keeper) and mindfulness apps (insight timer and smiling mind).

**Table 1.** Participant characteristics (N=208).

Characteristics	Values
Demographic and health information	
Age (years), mean (SD)	57.4 (10.8)
BMI (kg/m^2^), median (IQR)	26.0 (23.2‐30.75)
Comorbidity index, median (IQR)	1.0 (0.0‐1.0)
Location, n (%)	
Major cities in Australia	132 (63.5)
Inner regional	59 (28.4)
Outer regional/remote Australia	17 (8.2)
Marital status, n (%)	
Married, de facto, or living with partner	153 (73.6)
Separated, divorced, widowed, or single	53 (25.5)
Prefer not to say	2 (1)
Education, n (%)	
High school (Year 10 or 12)	16 (7.7)
Certificate or diploma (eg, TAFE or college)	55 (26.4)
University degree	137 (65.9)
Employment, n (%)	
Employed	103 (49.5)
Retired	66 (31.7)
Other	39 (18.8)
Aboriginal or Torres Strait Islander, n (%)	
No	206 (99)^a^
Language spoken at home, n (%)	
English	201 (96.6)
Other	7 (3.4)
Disease information	
Time since diagnosis (years), mean (SD)	2.2 (1.6)
Number of cardiotoxic treatments, median (IQR)	1.0 (1.0‐2.0)
Stage of disease, n (%)	
Stage I-II	148 (71.2)
Stage III-IV	57 (27.4)
Unsure	3 (1.4)
Treatment stage, n (%)	
Not yet started active treatment for cancer	2 (1)
Currently undergoing curative treatment	38 (18.3)
Completed curative treatment and in remission	119 (57.2)
Ongoing treatment to manage the disease	45 (21.6)
Other	4 (1.9)
Cardiotoxicity risk, n (%)	
High-risk^b^	94 (45.2)
Does not meet high-risk criteria	114 (54.8)
Knowledge of cardiac-related treatment side-effects, n (%)	
A little or not at all	120 (57.7)
Extremely or somewhat knowledgeable	88 (42.3)
Behavioral variables	
GLTEQ^c^ score (units), median (IQR)	45.0 (33.3‐62.0)
Resistance training (sessions per week), median (IQR)	2.0 (1.0‐4.0)
GLTEQ, n (%)	
Moderately active (<24 units)	14 (6.7)
Active (≥24 units)	194 (93.3)
Resistance training categories, n (%)	
Not meeting guidelines (<2 sessions per week)	117 (56.3)
Meeting guidelines (≥2 sessions per week)	91 (43.8)
Fitness tracker use, n (%)	
No, this does not interest me	16 (7.7)
No, but I would be open to trying	63 (30.3)
Yes, I have used a fitness tracker	129 (62)
Smartphone app use, n (%)	
No, this does not interest me	29 (13.9)
No, but I would be open to trying	92 (44.2)
Yes, I have used a fitness tracker	87 (41.8)

a No (n=1); Prefer not to say (n=1).

b Participants who had received a treatment associated with cardiotoxicity, had ≥2 risk factors for cardiovascular disease, or self-reported compromised cardiac function (see *Outcomes of Interest* section for additional detail).

c GLTEQ: Godin Leisure Time Exercise Questionnaire

### Likelihood of Participating in a Digitally Delivered Exercise Rehabilitation Program

Over 80% of participants reported being “extremely” or “quite a bit likely” to participate in a digitally delivered exercise program as part of their cancer treatment and recovery plan. When asked about specific timepoints, the majority of participants (>70%) were interested at any time, but particularly so in the after-treatment period (193/208, 93%) (Table 2).

**Table 2.** Likelihood of participating in a digitally delivered exercise rehabilitation program (N=208).

Timepoint	Not at all, n (%)	A little, n (%)	Somewhat, n (%)	Quite a bit, n (%)	Extremely, n (%)
In general *–* How likely is it that you would participate in a program like this if it was offered to you as part of your cancer treatment and recovery plan?	3 (1.4)	11 (5.3)	23 (11.1)	57 (27.4)	114 (54.8)
Before treatment – Exercising at this stage can help to prepare your body for treatment. It may also help you to stay more active during treatment.	12 (5.8)	14 (6.7)	34 (16.3)	38 (18.3)	110 (52.9)
During treatment – Exercising at this stage can help people to tolerate treatments and maintain quality of life.	6 (2.9)	15 (7.2)	36 (17.3)	68 (32.7)	83 (39.9)
After treatment – Exercising at this stage can help to overcome treatment side-effects, maintain quality of life and reduce risk of other health conditions.	1 (0.5)	2 (1.0)	12 (5.8)	47 (22.6)	146 (70.2)

### Characteristics Associated With Likelihood of Participating in a Digitally Delivered Exercise Rehabilitation Program

Results of the bivariate analyses are reported in Table S1 in Multimedia Appendix 2 and associations between demographic and health characteristics are reported in Table S2 in Multimedia Appendix 3. The demographic and health variables included in the final multivariate models were as follows: general uptake (BMI, education, number of cardiotoxic treatments, and location due to its potential theoretical importance in relation to digitally delivered services [age and employment were removed due to collinearity]); before (BMI, location, and education); during (comorbidity index and location [BMI was removed due to collinearity]); and after treatment (BMI, education, and location).

Results of the ordered logistic regression models are presented in Table 3. In the general uptake model, those living in an outer regional area reported higher odds for having a higher likelihood of participating in a digitally delivered exercise rehabilitation program, compared with those living in a major city (OR 4.05; 95% CI 1.04-15.81, *P*=.04). Having been treated with a higher number of cardiotoxic treatments was also associated with higher odds of being in a higher likelihood category (OR 1.42; 95% CI 1.02-1.96, *P*=.04). Whereas a higher BMI and a lower level of education (compared with university education) were associated with lower odds of reporting higher likelihood of uptake.

**Table 3.** Associations between participant characteristics and a higher category of likelihood to participate (N=208).

	General		Before		During		After	
	OR^a^ (95% CI)	*P* values	OR (95% CI)	*P* values	OR (95% CI)	*P* values	OR (95% CI)	*P* values
BMI	0.95 (0.91-0.99)	.02^b^	0.93 (0.89-0.98)	.003^b^	—^c^	—	0.94 (0.89-0.99)	.01^b^
Cardiotoxic treatments	1.42 (1.02-1.96)	.04^b^	—	—	—	—	—	—
Comorbidity index	—	—	—	—	0.65 (0.50-0.83)^d^	<.001^b^	—	—
Education								
University (n=137)	Ref^e^	Ref	Ref	Ref	—	—	Ref	Ref
Diploma (n=55)	0.37 (0.20-0.69)^d^	.002^b^	0.42 (0.23-0.78)^d^	.005^b^	—	—	0.30 (0.15-0.60)^d^	<.001^b^
High school (n=16)	0.52 (0.19-1.45)^d^	.21	0.48 (0.18-1.27)^d^	.14	—	—	0.38 (0.12-1.14)^d^	.085
Location								
Major city (n=132)	Ref	Ref	Ref	Ref	Ref	Ref	Ref	Ref
Inner regional (n=59)	1.01 (0.56-1.85)	.97	1.08 (0.60-1.98)	.79	0.76 (0.44-1.34)	.35	1.10 (0.55-2.19)	.79
Outer regional (n=17)	4.05 (1.04-15.81)^d^	.04^b^	4.84 (1.29-18.08)^d^	.02^b^	3.86 (1.39-10.73)^d^	.01^b^	8.57 (1.07-68.47)^d^	.04^b^

a OR: odds ratio

b Statistically significant: *P*<.05.

c N/A: not applicable; denotes variables that were not included in the multivariate models (see *Data Analyses* section for variable selection methods).

d Clinically relevant: OR<0.67 or >1.5.

e Ref: reference category.

Being from an outer regional area was associated with higher odds of reporting a higher likelihood of uptake before, during, and after treatment. However, ORs were not consistent across treatment phases. A higher BMI and a lower level of education (compared with university education) were associated with lower odds of being in a higher likelihood category both before and after treatment. As was a higher number of comorbid conditions during treatment.

### Other Intervention Topics of Interest

Participants were interested in a range of other topics to support their recovery or ongoing management of cancer. Table 4 lists interest in each topic, with weight maintenance, sleep, managing fatigue, and diet of interest to ≥50% of the sample. Mindfulness and stress reduction were also popular topics, with over one-third of participants reporting interest.

**Table 4.** Participant interest in other intervention topics as part of cancer treatment and recovery (N=208).

Topic	Values, n (%)
Diet	106 (51)
Weight maintenance	124 (59.6)
Smoking	0 (0)
Alcohol	23 (11.1)
Sleep	113 (54.3)
Falls prevention	18 (8.7)
Medication adherence	7 (3.4)
Stress	87 (41.8)
Memory	60 (28.8)
Managing fatigue	109 (52.4)
Mindfulness	95 (45.7)
Other^a^	18 (8.7)

a Other included mental health, lymphoedema massage, pain management, bone density management, self-examination, hot flushes, and chemo brain.

### Cost per Session

**Figure 1.**
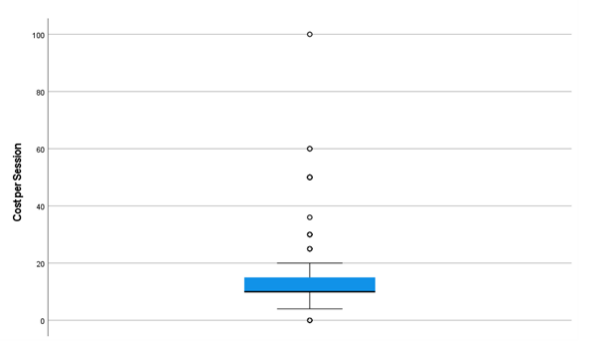
Amount willing or able to pay per session for an exercise program (n=206).

The distribution of participant responses regarding the amount willing or able to pay for an exercise program per session is displayed in Figure 1. The median amount was Aus $10 (US $6.9) per session (IQR Aus $10‐15). Few participants (21/208, 10%) could afford or were willing to pay over Aus $25 (US $17.25) per session, with most participants (163/208, 78%) reporting an amount of ≤Aus $15 (≤US $10.35).

## Discussion

### Principal Findings

This study examined the characteristics of breast cancer survivors associated with the likelihood of uptake of a digitally delivered cardiac exercise rehabilitation intervention. There was a high level of interest in uptake among the majority of participants across the treatment trajectory. There was also some evidence to support that living in an outer regional area and receiving more cardiotoxic treatments increased the likelihood of uptake further. Conversely, there was some evidence to suggest that patients with a higher BMI, more comorbid conditions, and lower education levels may be less likely to uptake the proposed intervention. Further investigation into these factors is critical for the delivery of a digital service model that is both effective and accessible.

The current findings are promising in that they suggest that the majority of breast cancer survivors are interested in uptaking a digitally delivered exercise rehabilitation intervention to improve cardiovascular fitness. This aligns with similar research in the area, with a high proportion of breast cancer survivors having reported an interest in participating in an exercise program or receiving physical activity counseling [30-32]. Breast cancer survivors have also reported high levels of interest (68%‐85%) in technology-supported exercise interventions specifically [16]. However, despite these promising levels of interest, physical activity levels typically decline following breast cancer diagnosis and remain low [33,34], thus suggesting that there is a need to develop intervention strategies and service delivery models that support breast cancer survivors to adopt and maintain regular activity.

The findings of this study provide some suggestions as to which patients (ie, those with a higher BMI, more comorbid conditions, and lower levels of education) may be less likely to uptake the proposed intervention. Recent research examining issues of digital health technology implementation in cancer care suggests that digital literacy may partially explain the current results [25]. Previous research has demonstrated that those with a lower education have lower digital literacy [35], which may impact the ability of this group to effectively engage in a digitally delivered intervention. Efforts to work collaboratively with those who may have lower digital literacy should be made to enhance the service delivery model for these groups, including its uptake, safety, feasibility, and effect [25]. Future research involving co-design processes and consumer involvement might be particularly beneficial for enhancing digital inclusion, which refers to the “access, affordability, usage, skills and relevance of digital technologies to individuals or groups” [25].

These results may also be reflective of general exercise barriers experienced by chronic disease populations (eg, those with risk factors of cardiotoxicity, such as a high BMI) [36]. Specifically, systematic review level evidence suggests that the primary barriers to participating in physical activity among people with a higher BMI include a lack of self-discipline or motivation, pain or discomfort, and a lack of access to equipment [36], all of which would also impact capabilities and resources for participating in a remotely-delivered intervention. Similarly, the presence of comorbidities has been identified as a barrier to physical activity among multiple cancer populations [37-39]. As such, the findings of the current study may be reflective of general barriers in this population, and so engagement with a digital intervention needs to address these barriers. This may be of particular importance among long-term breast cancer survivors, as a higher BMI and comorbidities such as high cholesterol, high blood pressure, diabetes, and previous cardiovascular disease are among the main risk factors for cardiotoxicity [40-42].

The secondary aim of this study was to evaluate other aspects of intervention delivery that would be useful for informing exercise oncology interventions among breast cancer survivors more broadly. Overall, the findings suggest the need for (1) multifaceted rehabilitation interventions that address multiple health behaviors (ie, diet and sleep as well as exercise), as well as mental health and symptom management, (2) better education for patients about potential cardiotoxic effects of treatment, and (3) interventions that are free or low cost for patients. Our findings relating to the need for multifaceted rehabilitation interventions mirror others [43]. In particular, the need for rehabilitation services to include multidisciplinary care that addresses not only physical well-being, but nutritional and psychological well-being and managing post-treatment challenges (eg, fatigue), as well as education regarding the potential heart-related side-effects of treatment [43].

Our findings in relation to service fees are novel, particularly considering the current chronic disease management plan in Australia and the affordability of private sessions. In Australia, the Chronic Disease Management initiative offers 5 government-rebated sessions with an allied health professional each year [44]. However, the current rebate (85% of the minimum service fee of ≈Aus $60 [≈US $41.4] [45,46]) leaves a payment gap above what has been reported as an acceptable cost per session by the current sample. Further, previous research suggests that those who are arguably the most disadvantaged (ie, those with a health care card or from a low socioeconomic or non-English speaking background) are less likely to be referred to physiotherapy services [47] and report being unable or unwilling to cover the payment gap attached to accessing allied health services [48,49]. In addition to continued advocacy at a government level to generate policies and funding models that support these services for people with chronic conditions, future research should aim to develop and evaluate service models that can provide effective, affordable, and equitable access to allied health services.

### Limitations

Several limitations should be considered alongside the results of the present analysis. First, the survey items posed to participants regarding their likelihood of uptake referred to their hypothetical interest and participation in digitally delivered cardiac exercise rehabilitation. As such, participant responses and findings of this analysis may or may not reflect real-life uptake. The cross-sectional design also precludes the exploration of longitudinal trajectories of the likelihood of uptake, and characteristics that may impact such trajectories. Additionally, considering that over half the sample had completed treatment at the time of the survey, items relating to participation pre- and during-treatment were collected retrospectively and as such, may be impacted by recall bias. The sample was also highly active (no sedentary participants and few insufficiently active), which may have biased the results and contributed to the high likelihood ratings across the cancer continuum and may not be representative of the broader breast cancer population. The recruitment strategy (web-based advertising) and study research questions resulted in it being more likely to appeal to women who were already active or who valued exercise. Web-based recruitment also limited participation to those with internet access (possibly excluding remote participants in areas without internet access) and those with sufficient digital literacy to complete a web-based survey (possibly excluding those who would be unable or unwilling to engage in digital services). This is of particular importance considering that the likelihood of participating in digital interventions was the primary outcome. Despite these limitations, a key strength of this analysis is its novelty in addressing the major service delivery challenges that have been observed in the cardiac rehabilitation setting, prior to developing an exercise rehabilitation intervention for cardiotoxicity following breast cancer treatment. Participants were also representative of the Australian breast cancer population in terms of age, geographical location, marital status, and health-related variables (eg, BMI) [50,51]. Further, this study investigated preferences regarding multidisciplinary rehabilitation topics and cost.

Findings from this research highlight several areas that warrant future investigation. Specifically, future research should explore the likelihood of uptake among a sample of sedentary or insufficiently active breast cancer survivors. This could provide valuable insight from those who potentially face the greatest barriers to exercise participation, as well as inform the development of more acceptable digital interventions. Additionally, findings suggest that patients with risk factors for cardiotoxicity (ie, those with a higher BMI and more comorbid conditions) may be less likely to uptake a digital exercise intervention. It would be of interest to investigate the likelihood of uptake for in-person programs in this subsample, to determine whether this is a delivery model issue or a more general exercise experience, and subsequently, what could a delivery model offer that would increase the likelihood of uptake.

### Conclusion

The findings of this study provide valuable knowledge regarding factors that influence the likelihood of uptake of a digitally delivered cardiac exercise rehabilitation intervention. These findings can inform future research and the development of intervention techniques that are critical to improving the delivery of a digital service model that is effective, equitable, and accessible to those at risk of cardiotoxicity following breast cancer treatment. Specifically, future research should aim to (1) enhance digital inclusion by collaboratively developing interventions that can be effectively engaged with by those who have lower digital literacy, (2) enhance engagement through the inclusion of techniques that address general barriers experienced by chronic disease populations, (3) incorporate multidisciplinary intervention topics that address multiple health behaviors, and (4) develop and evaluate the affordability of digital service delivery models.

## Supplementary material

10.2196/51536 Multimedia Appendix 1 Supplementary Document 1.

10.2196/51536 Multimedia Appendix 2 Supplementary Table 1.

10.2196/51536 Multimedia Appendix 3 Supplementary Table 2.

## Abbreviations

GLTEQ: Godin Leisure Time Exercise Questionnaire
HER-2: human epidermal growth factor receptor 2
OR: odds ratio
