# Supplementary material for: Demographics and Health Characteristics Associated With the Likelihood of Participating in Digitally Delivered Exercise Rehabilitation for Improving Heart Health Among Breast Cancer Survivors: Cross-Sectional Survey Study
Source: JMIR Cancer. 2024 Dec 16;10:e51536. doi: 10.2196/51536 (PMC11683507; doi:10.2196/51536)
Supplement: Multimedia Appendix 1 [file cancer-v10-e51536-s001.docx]

## Document S1

**EXERCISE CARE CLOSER TO HOME QUESTIONNAIRE**

**Eligibility**

**Question 1: Are you over the age of 18?**

- Yes
- No

**Question 2: Do you live in Australia?**

- Yes
- No

**Question 3: Have you been diagnosed with a haematological (Leukemia, Lymphoma, Myeloma) cancer in the past ﬁve years?**

- Yes
- No

**Section one – Exercise Support Experiences**

*Please answer each question as accurately as you can. At the end of the survey there will be an opportunity to provide any additional comments you would like to make and to leave you contact information (optional). Thank you for participating.*

**Question 1**: **Has anyone in your treatment team spoken to you about physical activity or exercising as part of your cancer treatment and recovery plan? Please select a response.**

- Yes
- No (Skip to question 3)

**Question 2:** **If yes: please specific which member of your care team (select all that apply):**

- Heamatologist
- Oncologist
- Surgeon
- Nurse
- GP
- Cardiologist
- Other (specify):

**Question 3: Have you received a referral to an exercise professional as part of your cancer treatment and recovery plan? Please select a response**.

- Yes
- No (Skip to question 8)

**Question 4: If yes: Please specify what type of professional you were referred to (please select all that apply):**

- Exercise physiologist
- Physiotherapist
- Personal gym trainer
- Other (specify):

**Question 5: Have you accessed the service(s) you were referred to?** **Please select a response.**

- Yes
- No, I do not feel I need it (Skip to question 8)
- No, I can not aﬀord it (Skip to question 8)
- No, It is too hard to get to (Skip to question 8)
- Not yet but I intend to (Skip to question 8)
- No, other reason (please specify) (Skip to question 8)

**If yes: Question 6: Do you feel like the professional(s) you were referred to adequately took your cancer diagnosis and any other health issues into account when providing you with exercise advice? Please select a response**

- Extremely so
- Quite a bit
- Somewhat
- A little
- Not at all

**Question 7: Do you feel like the professional(s) you were referred to adequately took your personal preferences, motivation level and resources (e.g., access to equipment and transport) into account when providing you with exercise advice? Please select a response**

- Extremely so
- Quite a bit
- Somewhat
- A little
- Not at all

**Question 8: Have you participated in a structured physical activity or exercise program as part of your cancer treatment or recovery plan? Please select a response**

- No (Skip to section two: exercise support preferences)
- Yes (please provide details. Of the program below e.g., who ran it and how long was it for?)

**Question 9: Have you continued with regular exercise or physical activity since completing the program? Please select a response**

- Very much so
- Quite a bit
- Somewhat
- A little
- Not at all

**Section two –Exercise Support Preferences**

**Question 1: If completing an exercise program for cancer recovery, which of the following formats would you prefer most?**

- Group-based exercise sessions
- Exercise sessions one-on-one with an instructor
- A program to complete on my own
- A mix of group-based sessions and exercises to do on my own
- A mix of one-on-one sessions with an instructor and exercises to do on my own

**Question 2: If there were an exercise program or activity that you liked, how much would you be willing or able to pay out of pocket in order to participate?**

Per session $:

Maximum per week $:

**Question 3: Where do you generally prefer to engage in exercise? Please select all that apply**

- Outside around my neighbourhood
- In my home
- At a community ﬁtness centre
- At a cancer centre
- At my treating hospital
- Other (specify):

We are interested in how likely you would be to participate in an exercise program for cancer recovery at different time-points. **Please rate how likely you think you would be to participate in a program during each of the phases described below.**When answering consider that exercise can have different benefits at different stages of your care, and that exercise programs are tailored to match your capabilities at a given time.

**Question 4: *Before treatment –* Exercising at this stage can help to prepare your body for treatment. It may also help you to stay more active during treatment. Please select a response**

- Extremely likely
- Quite a bit likely
- Somewhat likely
- A little likely
- Not at all likely

**Question 5: *During treatment –* Exercising at this stage can help people to tolerate treatments and maintain quality of life. Please select a response**

- Extremely likely
- Quite a bit likely
- Somewhat likely
- A little likely
- Not at all likely

**Question 6: *After treatment –* Exercising at this stage can help to overcome treatment side-effects, maintain quality of life and reduce risk of other health conditions. Please select a response**

- Extremely likely
- Quite a bit likely
- Somewhat likely
- A little likely
- Not at all likely

**Opinions about telehealth**

*These next questions are about the use of technology in home-based exercise programs.*

**As telehealth interventions can be delivered in a variety of ways please read the following real-world examples and describe how likely it is you would participate in this type of program if it was designed to support Heamatological cancer patients.**

**
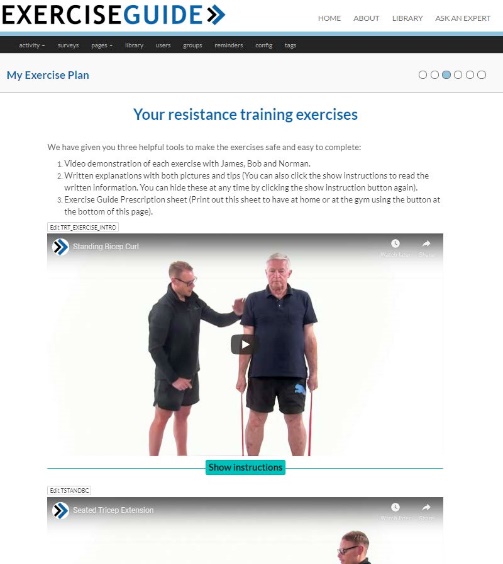
ExerciseGuide:** The ExerciseGuide website has been designed to support men with metastatic prostate cancer to participate in aerobic and strength exercises. An online assessment is completed by the user and an 8-week individualised exercise program is generated, which includes video instructions of how to complete each exercise. The user receives 2 telephone calls and weekly text messages from an accredited exercise physiologist to help them to complete the program.

**Question 7: How likely is it that you would participate in a program like ExerciseGuide if oﬀered to you as part of your cancer treatment and recovery plan?**

- Extremely
- Quite a bit
- Somewhat
- A little
- Not at all


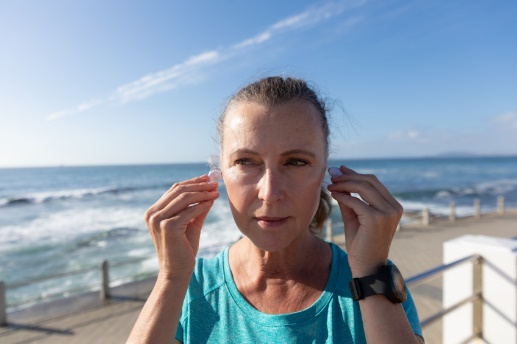
**REMOTE:** The Remote smartphone app has been designed to provide real-time supervised rehabilitation outside of a clinic-setting.  With the use of a heart rate sensor, mobile application and headphones users can complete aerobic exercise (e.g., walking, cycling) in their local neighbourhood or gym and receive real time feedback and encouragement from an exercise professional as if in a rehabilitation clinic. Users can also opt to select text messages to help keep them motivated

**Question 8: How likely is it that you would participate in a program like REMOTE if oﬀered to you as part of your cancer treatment and recovery plan? Please select a response**

- Extremely
- Quite a bit
- Somewhat
- A little
- Not at all


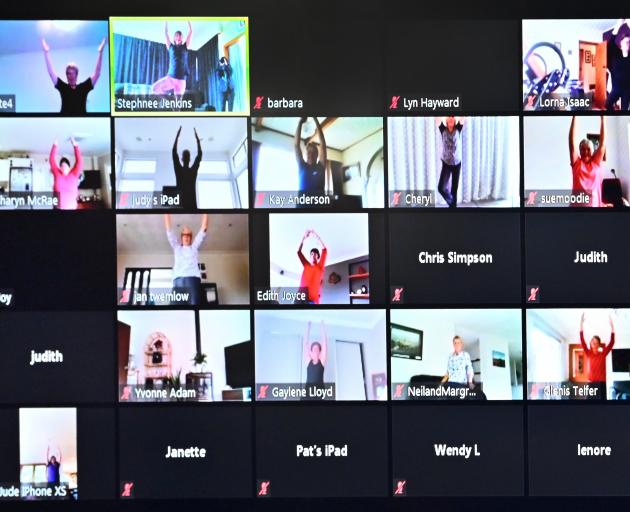
**Video Conferencing:**  In response to COVID, many exercise classes that were normally delivered face-to-face moved to online delivery. This typically involved video conferencing, with both the instructor and class attendee using a computer to see each other via video.

**Question 9: How likely is it that you would participate in an exercise program delivered via video conferencing if oﬀered to you as part of your cancer treatment and recovery plan? Please select a response**

- Extremely
- Quite a bit
- Somewhat
- A little
- Not at all

**Question 10: On the whole, how would you rate your confidence for participating in a telehealth exercise program? Please select a response**

- Extremely Confident
- Quite a bit Confident
- Somewhat Confident
- A little Confident
- Not at all Confident

**Question 11: Do you have any comments you would like to make about why you responded the way you did about your likelihood of participating in these programs?**

**Section three: Exercise motivation, confidence and tendencies**

**Question 1:** We are interested in the reasons underlying people’s decisions to engage, or not engage in exercise. **Using the scale below, please indicate to what extent each of the following items is true for you.** Please note that there are no right or wrong answers and no trick questions. We simply want to know how you feel personally about exercise. Your responses will be held in confidence and only used for our research purposes. **Please select only one response to the following questions.**

|  | Not true of me |  | Sometimes true |  | Very true |
| --- | --- | --- | --- | --- | --- |
| I exercise because other people say I should | O | O | O | O | O |
| I feel guilty when I do not exercise | O | O | O | O | O |
| I value the benefits of exercise | O | O | O | O | O |
| I exercise because it is fun | O | O | O | O | O |
| I do not see why I should have to exercise | O | O | O | O | O |
| I take part in exercise because my family/friends/partner say I should | O | O | O | O | O |
| I feel ashamed when I miss an exercise session | O | O | O | O | O |
| It is important to me to exercise regularly | O | O | O | O | O |
| I cannot see why I should bother exercising | O | O | O | O | O |
| I enjoy my exercise sessions | O | O | O | O | O |
| I exercise because others will not be pleased with me if I do not | O | O | O | O | O |
| I do not see the point in exercising | O | O | O | O | O |
| I feel like a failure when I have not exercised in a while | O | O | O | O | O |
| I think it is important to make the effort to exercise regularly | O | O | O | O | O |
| I find exercise a pleasurable activity | O | O | O | O | O |
| I feel under pressure from family/friends to exercise | O | O | O | O | O |
| I get restless if I do not exercise regularly | O | O | O | O | O |
| I get pleasure and satisfaction from participating in exercise | O | O | O | O | O |
| I think exercise is a waste of time | O | O | O | O | O |

**Question 2:** These questions look at how conﬁdent you are performing exercises and overcoming exercise challenges. **Please rate how conﬁdent you feel using the scale below for each of the items. Please select only one response to the following questions.**

|  | 0- not at all confident | 10 | 20 | 30 | 40 | 50 | 60 | 70 | 80 | 90 | 100 - completely confident |
| --- | --- | --- | --- | --- | --- | --- | --- | --- | --- | --- | --- |
| Complete exercises using proper technique | O | O | O | O | O | O | O | O | O | O | O |
| Follow direction to complete exercises | O | O | O | O | O | O | O | O | O | O | O |
| Perform all of the required movements | O | O | O | O | O | O | O | O | O | O | O |
| Exercise when you feel discomfort | O | O | O | O | O | O | O | O | O | O | O |
| Exercise when you lack energy | O | O | O | O | O | O | O | O | O | O | O |
| Exercise when you don't feel well | O | O | O | O | O | O | O | O | O | O | O |
| Include exercise in your daily routine | O | O | O | O | O | O | O | O | O | O | O |
| Consistently exercise three times per week | O | O | O | O | O | O | O | O | O | O | O |
| Arrange your schedule to include regular exercise | O | O | O | O | O | O | O | O | O | O | O |

**Question 3:** This question explores how automatic participating in exercise is for you. **To what extent do you agree with the following statements. Please only select one response to the following questions.**

|  | Strongly disagree | Disagree | Neutral | Agree | Strongly agree |
| --- | --- | --- | --- | --- | --- |
| Exercise is something I do automatically | O | O | O | O | O |
| Exercise is something I do without having to consciously remember | O | O | O | O | O |
| Exercise is something I do without thinking | O | O | O | O | O |
| Exercise is something I start doing before I realise I'm doing it | O | O | O | O | O |

**Question 4: Have you ever used a ﬁtness tracker (e.g. ﬁtbit, pedometer, smart watch) to monitor your exercise behaviour?**

- No, this does not interest me
- No, but I would be open to trying
- Yes

**Question 5: Have you ever used a smartphone app to help you change a health behaviour (e.g. exercise, diet, sleep)?**

- No, this does not interest me
- No, but I would be open to trying
- Yes, please specify which apps

*Thank you for answering the questions so far. The next few are designed to help us understand the types of people that are most interested in receiving lifestyle support and what types of support are of interest.*

**Section four – Health Behaviours**

**Question 1: During a typical 7-Day period, how many times on average do you do the following amount (times per week) of exercise for more than 15 minutes during your free time? Please put a number in the times per week column.**

Strenuous exercise (heart beats rapidly) (e.g., running, vigorous swimming)

Moderate exercise (not exhausting) (e.g., fast walking, easy swimming)

Mild/light exercise (minimal eﬀort) (e.g., bowling, golf, easy walking)

Resistance (strength) activities (e.g., yoga, weights, sit-ups)

**Question 2: During a typical 7-Day period, how many times on average do you do the following in regards to your food intake? Please put a number in the times per week column.**

Eat at least 2 serves of fruit per day? (1 Serve = 1 medium apple/orange/ banana or 2 small apricots/kiwi/plums or ½ cup diced/ canned fruit)

Eat at least 4 to 5 serves vegetables per day? (1 serve = ½ cup cooked vegetables or 1 cup salad leaves/ raw salad vegetables or ½ medium potato)

Eat at least 4 to 5 serves per day of wholegrains? (1 serve = 1 slice bread or ½ cup cooked rice/pasta/ quinoa/polenta/porridge or 2/3 cup cereal)

**Question 3: If available, would you like help with any of the following as part of a cancer treatment and recovery plan? (Please select all that apply)**

- Exercise
- Diet
- Weight maintenance
- Smoking
- Alcohol
- Sleep
- Falls prevention
- Medication adherence
- Stress
- Memory
- Managing fatigue
- Mindfulness
- Other (specify)

**Section five: Medical questions**

**Question 1: What type of heamatological cancer have you been diagnosed with?**

- Myeloma
- Leukaemia
- Non-Hodgkin lymphoma
- Hodgkin lymphoma

**Question 2: When were you ﬁrst diagnosed with heamatological cancer?**

Month:

Year:

**Question 3: What stage was your cancer when you were ﬁrst diagnosed? Please select a response**

- Stage 1 or A
- Stage 2 or B
- Stage 3 or C
- Stage 4
- Unsure

**Question 4: What type of cancer treatments are (/were) part of your treatment plan? Please select all that apply**

- Bone marrow or stem cell treatment
- Apheresis
- Chemotherapy, please specify type if known
- Surgery
- Radiation therapy
- Other, please specify
- I don’t know

**Question 5: *If yes to bone marrow*: How many times have you had a bone marrow or stem cell transplant as part of your treatment? Please select a response**

- None, but I am scheduled to have one.
- Once
- Twice
- Three or more times

**Question 6: Which of the following best describes your circumstances? Please select a response**

- I have not yet started active treatment for cancer (e.g. chemotherapy, radiotherapy, surgery, stem cell transplant)
- I am currently undergoing curative treatment
- I have completed treatment and am in remission
- I am having on-going treatment to manage the disease
- Other, please specify

**Question 7: What type of hospital is managing your care? Please select a response**

- Public
- Private

**Question 8: Has a doctor ever told you that you have any of the following health conditions? Please select all that apply**

- High cholesterol
- High blood pressure
- Diabetes type 1 or 2
- Congestive heart failure (or heart disease)
- Heart attack (myocardial infarct)
- Stroke or TIA
- Depression or anxiety
- Other, please specify

**Question 9: What is your height?**

in cm

**Question 10: What is your current weight?**

in kg

**Question 11: Have you had any weight loss in the last six months?**

- No
- Yes, please specify how much (kg)

and if yes:

- Intentional
- Unintentional

**Question 12: In general, would you say your health is. Please select a response**

- Poor
- Fair
- Good
- Very good
- Excellent

**Section six: Demographics**

**Question 1: What is your current age?**

**Question 2: What is your home postcode?**

**Question 3: What gender do you identify as?**

- Female
- Male
- Prefer not to say
- Please specify

**Question 4: What is your current relationship status?**

- Married, de-factor or living with partner
- Separated or divorced
- Widowed
- Single
- Prefer not to say

**Question 5: What is your highest level of education?**

- Primary school (Year 6)
- High school (Year 10 or 12)
- Certiﬁcate or diploma (e.g., TAFE or college)
- University degree
- Other, please specify

**Question 6: Are you of Aboriginal or Torres Strait Islander origin?**

- No
- Yes, Aboriginal
- Yes, Torres Strait Islander
- Yes, both Aboriginal and Torres Strait Islander
- Prefer not to say

**Question 7: Do you speak any language at home other than English?**

- No
- Yes, please specify

**Question 8: What best describes your employment at this time?**

- Full-time
- Part-time
- Casual
- Retired
- Household/caring duties
- Volunteer
- On leave without pay
- On leave with pay
- Unable to work due to diagnosis/treatment, receiving pension
- Unemployed
- Other, please specify

**Question 9: Do you have private health insurance?**

- Yes
- No

**Question 10: Do you hold a concession card?**

- Yes
- No

**Question 11: Do you have any additional comments you would like to make? (Optional)**
