# Supplementary material for: Demographics and Health Characteristics Associated With the Likelihood of Participating in Digitally Delivered Exercise Rehabilitation for Improving Heart Health Among Breast Cancer Survivors: Cross-Sectional Survey Study
Source: JMIR Cancer. 2024 Dec 16;10:e51536. doi: 10.2196/51536 (PMC11683507; doi:10.2196/51536)
Supplement: Multimedia Appendix 2 [file cancer-v10-e51536-s002.docx]

## Table S1. Bivariate Associations of Demographic and Health Variables with Likelihood of Uptake

|  | **Generally** | | **Before** | | **During** | **After** |
| --- | --- | --- | --- | --- | --- | --- |
| **Demographic & Health Information** | **B (95% CI), *P*** | **B (95% CI), *P*** | | **B (95% CI), *P*** | | **B (95% CI), *P*** |
| Age, years | 0.959 (0.934, 0.984), .001 | 0.911 (0.968, 1.015), .468 | | 0.996 (0.973, 1.019), .719 | | 0.987 (0.960, 1.015), .368 |
| BMI, kg/m^2^ | 0.949 (0.908, 0.908), .018 | 0.932 (0.889, 0.976), .003 | | 0.952 (0.912, 0.994), .025 | | 0.937 (0.894, 0.983), .008 |
| Comorbidity Index | 0.889 (0.691, 0.691), .360 | 0.819 (0.640, 1.048), .113 | | 0.668 (0.521, 0.855), .001 | | 0.779 (0.586, 1.035), .085 |
| Location | *P*=.110 | *P*=.064 | | *P*=.026 | | *P*=.099 |
| Major Cities Australia | Ref | Ref | | Ref | | Ref |
| Inner Regional | 0.763 (0.431, 1.349), .352 | 0.827 (0.466, 1.469), .517 | | 0.776 (0.443, 1.359), .375 | | 0.744 (0.392, 1.413), .367 |
| Outer Regional/Remote Australia | 3.168 (0.858, 11.699), .084 | 4.102 (1.131, 14.883), .032 | | 3.383 (1.235, 9.267), .018 | | 6.937 (0.891, 54.009), .064 |
| Marital Status |  |  | |  | |  |
| Separated, divorced, widowed, single (prefer not to say) | Ref | Ref | | Ref | | Ref |
| Married, de-facto, or living with partner | 1.321 (0.738, 2.364), .349 | 0.878 (0.494, 1.561), .658 | | 0.731 (0.411, 1.300), .286 | | 1.149 (0.599, 2.207), .676 |
| Education | *P*=.002 | *P*=.010 | | *P*=.065 | | *P*=<.001 |
| University Degree | Ref | Ref | | Ref | | Ref |
| Certificate or Diploma (e.g., TAFE or College) | 0.345 (0.190, 0.625), <.001 | 0.415 (0.232, 0.743), .003 | | 0.510 (0.289, 0.899), .020 | | 0.289 (0.150, 0.557), <.001 |
| High School (Year 10 or 12) | 0.516 (0.188, 1.412), .197 | 0.530 (0.202, 1.388), .196 | | 0.719 (0.266, 1.941), .515 | | 0.426 (0.144, 1.259), .123 |
| Employment | *P*=.003 | *P=*.337 | | *P*=.885 | | *P*=.946 |
| Employed | Ref | Ref | | Ref | | Ref |
| Retired | 0.425 (0.233, 0.776), .005 | 0.719 (0.399, 1.296), .273 | | 0.952 (0.543, 1.669), .864 | | 0.895 (0.463, 1.730), .741 |
| Other | 0.349 (0.171, 0.710), .004 | 0.635 (0.324, 1.243), .185 | | 0.842 (0.426, 1.663), .621 | | 0.944 (0.418, 2.133), .891 |
| **Disease Information** |  |  | |  | |  |
| Time since diagnosis, years | 0.962 (0.811, 1.142), .662 | 0.941 (0.800, 1.108), .466 | | 0.897 (0.765, 1.052), .182 | | 0.965 (0.799, 1.166), .712 |
| Number of cardiotoxic treatments | 1.368 (1.003, 1.864), .048 | 1.197 (0.887, 1.615), .239 | | 0.934 (0.705, 1.237), .632 | | 0.181 (0.838, 1.665), .343 |
| Stage of Disease (n=3 excluded) |  |  | |  | |  |
| Stage I-II | Ref | Ref | | Ref | | Ref |
| Stage III-IV | 1.080 (0.608, 1.917), .793 | 1.647 (0.915, 2.962), .096 | | 1.016 (0.587, 1.759), .954 | | 1.093 (0.574, 2.082), .786 |
| Treatment Stage (n=6 excluded) | *P*=.266 | *P*=.961 | | *P*=.420 | | *P*=.677 |
| Currently undergoing curative treatment | Ref | Ref | | Ref | | Ref |
| Completed curative treatment and in remission | 1.303 (0.648, 2.624), .458 | 0.952 (0.473, 1.917), .890 | | 0.737 (0.374, 1.450), .376 | | 0.763 (0.329, 1.770), .529 |
| Ongoing treatment to manage the disease | 1.984 (0.854, 4.609), .111 | 0.890 (0.388, 2.039), .783 | | 1.077 (0.480, 2.418), .858 | | 0.649 (0.249, 1.695), .378 |
| Cardiotoxicity Risk |  |  | |  | |  |
| Does not meet high risk criteria | Ref | Ref | | Ref | | Ref |
| High Risk | 0.766 (0.454, 1.292), .317 | 1.158 (0.690, 1.944), .578 | | 0.957 (0.580, 1.578), .864 | | 1.031 (0.572, 1.860), .919 |
| Knowledge of cardiac-related treatment side-effects |  |  | |  | |  |
| A little/Not at all | Ref | Ref | | Ref | | Ref |
| Extremely/Somewhat knowledgeable | 1.456 (0.853, 2.484), .168 | 1.491 (0.878, 2.531), .139 | | 1.383 (0.835, 2.291), .208 | | 1.266 (0.695, 2.306), .442 |

CI: confidence interval.
