# Supplementary material for: Demographics and Health Characteristics Associated With the Likelihood of Participating in Digitally Delivered Exercise Rehabilitation for Improving Heart Health Among Breast Cancer Survivors: Cross-Sectional Survey Study
Source: JMIR Cancer. 2024 Dec 16;10:e51536. doi: 10.2196/51536 (PMC11683507; doi:10.2196/51536)
Supplement: Multimedia Appendix 3 [file cancer-v10-e51536-s003.docx]

## Table S2. Correlations

|  | Age  (Continuous) | Body Mass Index  (Continuous) | Comorbidity  (Continuous) | Location  (Categorical) | Education  (Categorical) | Employment  (Categorical) | Cardiotoxic Treatment  (Continuous) |
| --- | --- | --- | --- | --- | --- | --- | --- |
| Age  (Continuous) |  |  |  |  |  |  |  |
| Body Mass Index  (Continuous) | -.029 (.676); -.018 (.792) |  |  |  |  |  |  |
| Comorbidity  (Continuous) | .241 (<.001); .232 (<.001) | .290 (<.001); .245 (<.001) |  |  |  |  |  |
| Location  (Categorical) | .932 | .238 | .850 |  |  |  |  |
| Education  (Categorical) | .008 | .479 | .003 | .054 |  |  |  |
| Employment  (Categorical) | .000 | .220 | .020 | .062 | <.001 |  |  |
| Cardiotoxic Treatment  (Continuous) | -.196 (.004) -.201 (.004) | .044 (.527); .026 (.713) | -.022 (.753); -.002 (.977) | .598 | .459 | .060 |  |

Continuous x Continuous: Pearson Correlation (*P*-value); Spearman Correlation (*P*-value)

Continuous x Categorical: Regression *P*-value

Categorical x Categorical: Pearson Chi Square (*P*-value)
